# Association of Intraindividual Difference in Cystatin C and Creatinine Estimated Glomerular Filtration Rate With Diabetes

**DOI:** 10.1155/jdr/9335243

**Published:** 2025-11-13

**Authors:** Lingyu Zhang, Yongjiang Yu, Yunyun Zhao, Xiuge Wang

**Affiliations:** ^1^Changchun University of Chinese Medicine, Changchun, Jilin, China; ^2^Department of Endocrinology, The Affiliated Hospital to Changchun University of Chinese Medicine, Changchun, Jilin, China

**Keywords:** CHARLS, creatinine, cystatin C, diabetes mellitus, eGFR

## Abstract

**Introduction:**

Declined renal function is closely linked to diabetes. However, it remains unclear whether the intraindividual difference between cystatin C- and creatinine-based estimated glomerular filtration rates (eGFRdiff) is associated with diabetes. This study was aimed at examining the association between eGFRdiff and prevalent diabetes in a nationally representative cohort of Chinese adults.

**Methods:**

We analyzed data from 11,869 adults aged ≥ 45 years participating in the China Health and Retirement Longitudinal Study (CHARLS), including 2279 individuals with diabetes. We calculated eGFRdiff as the absolute difference between cystatin C- and creatinine-based eGFR levels. Multivariable logistic regression and restricted cubic spline models were used to assess the association between eGFRdiff and prevalent diabetes. Subgroup analyses were conducted on sex, body mass index, hypertension status, and creatinine-based eGFR status.

**Results:**

The mean participant age was 60.3 (±9.6) years, and 53.5% were female. Participants were categorized into three groups based on eGFRdiff: midrange (−15 to 15 mL/min/1.73 m^2^, 67.9%), negative (<−15, 22.7%), and positive (> 15, 9.4%). Compared to the midrange group, individuals in the negative eGFRdiff group had a significantly higher odds of diabetes, even after adjusting for the creatinine-based eGFR (OR: 1.21, 95% CI: 1.08–1.36) and cystatin C-based eGFR (OR: 1.32, 95% CI: 1.16–1.50).

**Conclusion:**

In this large, community-based population, a negative eGFRdiff—where cystatin C-based eGFR is substantially lower than creatinine-based eGFR—is associated with a higher prevalence of diabetes, independent of overall kidney function. These findings suggest that eGFRdiff may serve as a novel marker for metabolic status.

## 1. Introduction

Diabetes has become a major public health concern, affecting over 500 million individuals worldwide, with China experiencing the highest national burden [1, 2]. As a complex metabolic disorder, diabetes arises from the interplay of genetic, lifestyle, and physiological factors, underscoring the need for refined risk assessment from multiple domains to support early detection and intervention strategies [3, 4].

Renal function decline is a recognized complication and contributor to diabetes, with ample evidence indicating a bidirectional relationship between impaired kidney function and diabetes onset [5, 6]. Renal impairment in diabetes is often attributed to microvascular injury affecting the kidneys. The estimated glomerular filtration rate (eGFR) is widely used to assess kidney function. Traditionally, eGFR is calculated using serum creatinine (eGFRcr), which, while clinically established, can be influenced by factors such as muscle mass, physical activity, and dietary protein intake [7]. Cystatin C has emerged as an alternative biomarker that may offer a more consistent reflection of glomerular filtration, being less susceptible to the nonrenal influences affecting creatinine [8], such as physical activity, muscle mass, and diet [9]. In recognition of this, clinical guidelines including those from the Kidney Disease Improving Global Outcomes (KDIGO) recommend incorporating cystatin C-based eGFR (eGFRcys) into practice for improved accuracy [10]. Due to the different biological properties and influencing factors, discrepancies often arise between eGFRcys and eGFRcr values. This difference—referred to as eGFRdiff—may indicate the influence of non-GFR-related determinants [11]. A large eGFRdiff may reflect the existence of non-GFR determinants [12]. With increasing adoption of cystatin C in clinical settings, a growing number of individuals may present with notable eGFRdiff, yet the health implications of this discordance remain insufficiently understood [13].

While eGFRdiff has been associated with various health outcomes, such as kidney disease [11], cardiovascular disease [14], heart failure [15], frailty [16], physical function [17], and cognition [18], its relationship with diabetes has not been well characterized—especially in aging populations where both renal and metabolic risks converge.

To address this knowledge gap, we utilized nationally representative data from the China Health and Retirement Longitudinal Study (CHARLS) to investigate the association between eGFRdiff and the prevalence of diabetes. We hypothesized that a large negative discordance in eGFRdiff is significantly associated with diabetes prevalence, independent of the absolute values of eGFRcr or eGFRcys.

## 2. Materials and Methods

### 2.1. Study Population

CHARLS is a nationally representative cohort that collected data from adults aged 45 years and older to support research on health and aging in China (http://charls.pku.edu.cn/). Participants were recruited using a multistage, stratified, probability-proportional-to-size sampling design, covering 28 provinces, including over 10,000 households across rural and urban regions. Detailed information on the cohort methodology has been previously published [19]. All participants provided written informed consent prior to participation. Ethical approval for the CHARLS study was granted by the Biomedical Ethics Review Committee of Peking University.

Face-to-face interviews were conducted using computer-assisted personal interviewing (CAPI), gathering data on demographics, health status, and lifestyle behaviors [20]. Physical examinations and venous blood collection were also conducted. For this analysis, we used data from the 2015 survey wave. From 13,420 participants with blood examination data, we excluded individuals if they (1) had missing data on cystatin C or creatinine (*n* = 814), (2) were younger than 45 years (*n* = 557), or (3) reported a prior cancer diagnosis at or before the 2015 survey (*n* = 180).

### 2.2. Data Collection

Sociodemographic variables included age, sex, educational attainment (primary or below, secondary, and tertiary), and marital status (married or others). Lifestyle factors, such as smoking (never, former, and current), were also recorded. Body weight and height were measured, and body mass index (BMI) was calculated. Based on BMI, individuals were categorized as lean (BMI < 24.0 kg/m^2^), overweight (BMI ≥ 24.0 kg/m^2^), or obese (BMI ≥ 28.0 kg/m^2^) according to Chinese criteria.

Prevalent diabetes was defined based on at least one of the following: fasting blood glucose ≥ 7.0 mmol/L, hemoglobin A1c (HbA1c) ≥ 6.5%, or self-reported diagnosis of diabetes, following the American Diabetes Association guidelines [21].

### 2.3. Exposure

Venous blood samples were analyzed in a certified laboratory. Serum cystatin C (milligrams per liter) was measured using a particle-enhanced turbidimetric assay, and the coefficient of variation (CV) was less than 5% for the measured platform. Serum creatinine (milligrams per deciliter) was measured using the rate-blanked and compensated Jaffe method. The cystatin C- and creatinine-based eGFR values were calculated based on the 2021 CKD-EPI equations [22]. We calculated eGFRdiff as the absolute difference between cystatin C- and creatinine-based eGFR levels. Participants were categorized into three groups according to eGFRdiff levels, that is, midrange (−15 to 15 mL/min/1.73 m^2^), negative (< −15 mL/min/1.73 m^2^), and positive (> 15 mL/min/1.73 m^2^), consistent with prior literature [23].

### 2.4. Statistical Analysis

Descriptive statistics were used to summarize baseline characteristics overall and stratified by diabetes status and eGFRdiff categories. Means (standard deviations) were used for continuous variables, and frequencies (percentages) were used for categorical variables.

Binomial logistic regression models were applied to assess the association between eGFRdiff groups and prevalent diabetes. We adjusted for either eGFRcys or eGFRcr to explore the independent effect of eGFRdiff (Model 1). Considering the clinical significance and aligning with existing studies, adjusted covariates in the full model included age, sex, education, marital status, smoking status, alcohol consumption, BMI categories, and hypertension (Model 2).

To evaluate nonlinear associations, restricted cubic spline models were constructed using knots at the 5th, 50th, and 95th percentiles of eGFRdiff values. Subgroup analyses were conducted by sex, obesity status, hypertension, and eGFRcr levels. All statistical tests were two-sided, and *p* values < 0.05 were considered statistically significant. Analyses were performed using R software (Version 4.1.0).

## 3. Results

**Figure 1:**
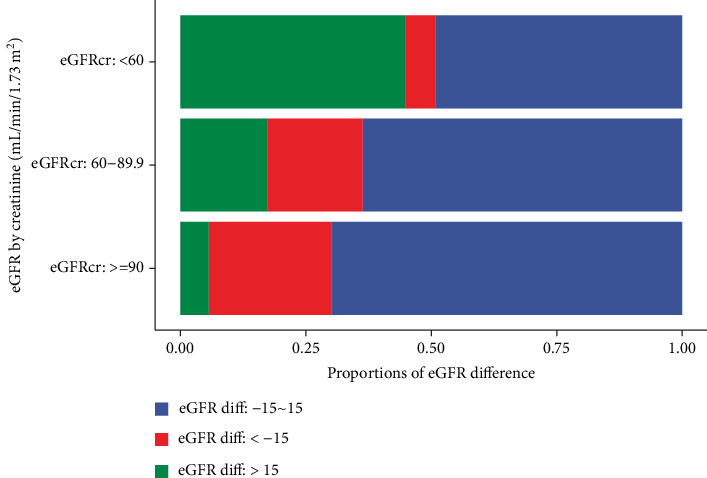
Distribution of eGFR difference categories stratified by creatinine-based eGFR levels. Abbreviation: eGFR, estimated glomerular filtration rate. The unit of eGFR was mL/min/1.73 m^2^ estimated by CKD-EPI equations. The eGFRdiff was defined as the absolute difference between cystatin C- and creatinine-based eGFR levels.

Among 11,869 CHARLS participants, 6355 (53.5%) were female, and the mean (SD) age was 60.3 (9.6) years. A total of 2279 participants (19.2%) had diabetes at the survey (Table 1). Those with diabetes were older and had higher BMI levels at the survey point. Table S1 shows the characteristics stratified by the eGFRdiff levels. Those in the negative eGFRdiff group tended to be older, more likely to be female, and had higher BMI levels. As presented in Figure 1, the proportions of the negative eGFRdiff group decreased along with declined eGFR levels by creatinine.

**Figure 2:**
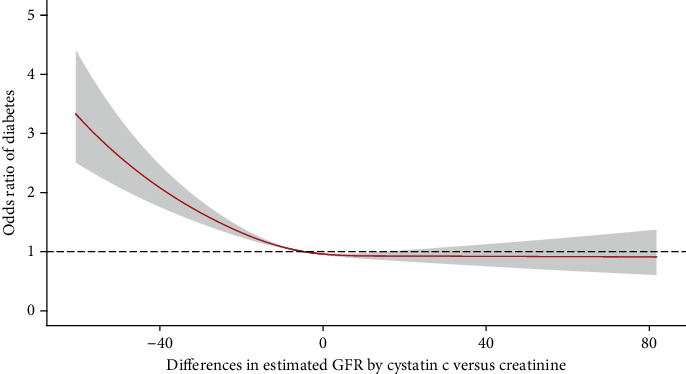
Dose–responsive relationship of eGFR difference with prevalence of diabetes. Abbreviation: eGFR, estimated glomerular filtration rate. The unit of eGFR was mL/min/1.73 m^2^ estimated by CKD-EPI equations. The eGFRdiff was defined as the absolute difference between cystatin C- and creatinine-based eGFR levels. The relationship was estimated using restricted cubic splines with knots at the 5th, 50th, and 95th percentiles of the distribution of eGFRdiff levels. Analysis was adjusted for age, sex, education degree, marriage status, smoking status, current drinking, BMI group, hypertension, and eGFRcr levels.

The associations between diabetes and declined eGFR calculated by cystatin C and creatinine were observed in this analysis (Table S2). There was a distinctly increased odds of diabetes corresponding to negative eGFRdiff values (Figure 2). In the fully adjusted model, those in the negative group had a significantly higher odds of diabetes after adjusting for the creatinine-based eGFR (OR: 1.21, 95% CI: 1.08–1.36) compared to the midrange group (Table 2). The association between eGFRdiff and diabetes was also significant after adjusting for cystatin C-based eGFR (OR: 1.32, 95% CI: 1.16–1.50). In unadjusted models, people in the positive eGFRdiff group had a decreased prevalence of diabetes, which was attenuated to insignificant in the adjusted models.

We performed subgroup analyses in terms of sex, obesity, hypertension, and eGFRcr level. The results were almost consistent when stratified by these important factors (Table 3). Among those of eGFRcr < 90 mL/min/1.73 m^2^, the OR (95% CI) for negative eGFRdiff group was 1.47 (1.15–1.86), while the OR (95% CI) for negative eGFRdiff group among those of normal eGFRcr values was 1.20 (1.06–1.36).

## 4. Discussion

In this nationally representative study of Chinese adults aged 45 years and older, we found that the intraindividual difference between cystatin C- and creatinine-based eGFR was significantly associated with the prevalence of diabetes. This association remained robust even after adjusting for either eGFRcys or eGFRcr, suggesting that eGFRdiff may provide additional metabolic health insights beyond conventional renal function markers. Our findings were consistent across key subgroups defined by sex, obesity, hypertension status, and baseline renal function status. To our knowledge, this is the first study to investigate the relevance of eGFRdiff in relation to diabetes prevalence in a general population.

The clinical use of cystatin C has led to the recognition that discrepancies often exist between eGFRcys and eGFRcr. Individuals with significantly lower eGFRcys compared to eGFRcr—resulting in a large negative eGFRdiff—have previously been shown to be at higher risk of adverse health outcomes, including frailty, functional decline, and cardiovascular events [24, 25]. For instance, data from the Systolic Blood Pressure Intervention Trial (SPRINT) indicated that eGFRdiff may serve as a surrogate for age-related physiological decline, with positive values potentially reflecting preserved muscle mass and resilience to frailty [16]. Similarly, an analysis using CHARLS data linked large eGFR discrepancies to deteriorating physical function over time [17]. The frailty index includes multiple dimensions, such as medical history of diabetes and energy level, which are closely related to diabetes status. The relationship of eGFRdiff with cardiovascular health and outcomes is also indicated. A study reported that a large eGFRdiff is associated with a higher risk of major adverse cardiovascular events and coronary artery calcification progression among people with chronic kidney disease [26]. Patients with kidney disease and a large eGFRdiff are facing a higher risk of heart failure and mortality [11, 15]. These observations support the notion that eGFRdiff may integrate signals from aging, inflammation, and metabolic dysfunction—factors closely related to diabetes pathogenesis and glucose metabolism.

Our findings expand on this evidence by demonstrating that individuals with negative eGFRdiff values were more likely to have prevalent diabetes, independent of conventional eGFR measures. In our cohort, approximately 22.7% of participants exhibited large negative differences, and these individuals were disproportionately older and female—patterns consistent with earlier reports [24]. In our analysis, the declined renal function measured by eGFRcr and eGFRcys is both related to the prevalence of diabetes. The novel implication is that the association between eGFRdiff and diabetes remains after adjusting for eGFRcr or eGFRcys levels, indicating that eGFRdiff could capture specific biological signals other than eGFRcr and eGFRcys themselves.

Emerging evidence also links eGFRdiff to the prognosis and health outcomes of individuals with diabetes. A population-based cohort study among adults with diabetes found that a larger eGFRdiff was significantly associated with a higher risk of diabetic microvascular complications, such as retinopathy and nephropathy [27]. In addition, a large cohort study reported that eGFRdiff represented a marker of adverse events for diabetes and was more strongly associated with cardiovascular events and mortality in individuals with diabetes [28].

There are possible explanations linking eGFRdiff and diabetes. Biologically, this discordance may relate to the distinct pathways influencing cystatin C and creatinine concentrations. Cystatin C is less influenced by muscle mass, diet, or physical activity—factors that can confound creatinine-based estimates—making it a potentially more stable marker of renal function and systemic inflammation [29]. Also, insulin resistance may trigger the renal damages [30] that could be early captured by higher cystatin C levels. Studies also suggested that cystatin C is a better biomarker to reflect the diabetes status [31]. Furthermore, recent Mendelian randomization analyses support a potential causal role of cystatin C in the development of diabetic complications, whereas such associations were not found for creatinine [32].

Several limitations merit consideration. First, due to the cross-sectional design, we cannot infer temporality or causality between eGFRdiff and diabetes. Second, the analysis was limited to middle-aged and older Chinese adults, and whether these findings generalize to other populations or age groups remains to be determined. Third, although we adjusted for a broad range of confounders, residual confounding by unmeasured factors cannot be ruled out.

In conclusion, our findings indicate that a large negative difference between eGFRcys and eGFRcr (i.e., negative eGFRdiff) was significantly associated with increased odds of having diabetes, independent of either eGFR measure alone. Incorporating eGFRdiff into clinical evaluations may improve the early detection of diabetes and guide more tailored prevention strategies.

## Figures and Tables

**Table 1:** Characteristics of 11,869 individuals according to prevalent diabetes.

	**Overall**	**No diabetes**	**Diabetes**	**p** ** value**
Participants, no.	11,869	9590	2279	
Age (years)				
Continuous	60.3 (9.6)	59.9 (9.6)	62.0 (9.3)	< 0.001
45–54	3853 (32.5)	3290 (34.3)	563 (24.7)	< 0.001
55–64	4179 (35.2)	3348 (34.9)	831 (36.5)	
65–74	2845 (24.0)	2188 (22.8)	657 (28.8)	
≥ 75	992 (8.4)	764 (8.0)	228 (10.0)	
Female, *n* (%)	6355 (53.5)	5084 (53.0)	1271 (55.8)	0.019
Married, *n* (%)	10,382 (87.5)	8411 (87.7)	1971 (86.5)	0.122
Educational level, *n* (%)				0.104
Primary or below	6786 (57.2)	5448 (56.8)	1338 (58.7)	
Middle or tertiary	5083 (42.8)	4142 (43.2)	941 (41.3)	
BMI (kg/m^2^)				
Continuous	24.0 (3.8)	23.7 (3.6)	25.3 (3.9)	< 0.001
< 23.9	6155 (51.9)	5326 (55.5)	829 (36.4)	< 0.001
24–27.9	3900 (32.9)	3002 (31.3)	898 (39.4)	
≥ 28	1814 (15.3)	1262 (13.2)	552 (24.2)	
Smoking status, *n* (%)				< 0.001
Never	7077 (59.6)	5703 (59.5)	1374 (60.3)	
Current	3277 (27.6)	2710 (28.3)	567 (24.9)	
Quit	1515 (12.8)	1177 (12.3)	338 (14.8)	
Current drinking, *n* (%)	4188 (35.3)	3484 (36.3)	704 (30.9)	< 0.001

*Note:* Data are presented as the mean (SD) or number (percent), as appropriate. BMI was calculated as weight in kilograms divided by height in meters squared.

Abbreviations: BMI, body mass index; SD, standard deviation.

**Table 2:** Associations of eGFR difference by cystatin C versus creatinine with prevalent diabetes.

	**No. diabetes/ ** **N**	**Model 1**		**Model 2**	
		**OR (95% CI)**	**p** ** value**	**OR (95% CI)**	**p** ** value**
Panel 1					
eGFRdiff: −15~15	1443/8057	Ref			
< −15	657/2699	1.49 (1.34–1.65)	< 0.001	1.21 (1.08–1.36)	0.001
> 15	179/1113	0.81 (0.67–0.96)	0.016	1.03 (0.85–1.23)	0.779
Panel 2					
eGFRdiff: −15~15	1443/8057	Ref			
< −15	657/2699	1.34 (1.19–1.52)	< 0.001	1.32 (1.16–1.50)	< 0.001
> 15	179/1113	0.90 (0.76–1.07)	0.233	0.97 (0.81–1.16)	0.765

*Note:* The unit of eGFR was mL/min/1.73 m^2^ estimated by CKD-EPI equations. The eGFRdiff was defined as the absolute difference between cystatin C- and creatinine-based eGFR levels. Model 1 was adjusted for the eGFR levels calculated based on creatinine (Panel 1) or cystatin C (Panel 2). Model 2 was further adjusted for age, sex, education, marriage status, smoking status, current drinking, BMI group, and hypertension.

Abbreviations: CI, confidence interval; eGFR, estimated glomerular filtration rate; OR, odds ratio.

**Table 3:** Associations of eGFRdiff categories with prevalent diabetes, stratified by subgroup.

**Subgroup**	**eGFRdiff: < −15**		**eGFRdiff: > 15**	
	**OR (95% CI)**	**p** ** value**	**OR (95% CI)**	**p** ** value**
Male	1.22 (1.00–1.46)	0.042	0.83 (0.68–1.02)	0.073
Female	1.23 (1.07–1.42)	0.003	0.98 (0.65–1.46)	0.937
Nonobesity	1.22 (1.07–1.39)	0.002	0.95 (0.77–1.16)	0.589
Obesity	1.29 (1.02–1.63)	0.031	1.47 (0.92–2.31)	0.101
No hypertension	1.33 (1.15–1.53)	< 0.001	0.90 (0.72–1.11)	0.329
Hypertension	1.26 (1.06–1.49)	0.009	1.01 (0.72–1.40)	0.974
*eGFRcr* ≥ 90	1.20 (1.06–1.36)	0.005	1.03 (0.79–1.33)	0.815
*eGFRcr* < 90	1.47 (1.15–1.86)	0.002	0.84 (0.66–1.07)	0.169

*Note:* The unit of eGFR was mL/min/1.73 m^2^ estimated by CKD-EPI equations. The eGFRdiff was defined as the absolute difference between cystatin C- and creatinine-based eGFR levels. Age, sex, education degree, marriage status, smoking status, current drinking, BMI group, hypertension, and eGFRcr levels were adjusted.

Abbreviations: CI, confidence interval; eGFR, estimated glomerular filtration rate; OR, odds ratio.

## Data Availability

The original datasets are publicly available online, and the final datasets generated for this study are available upon request from the corresponding authors.

## Supporting Information

Supporting Information Additional supporting information can be found online in the Supporting Information section. Table S1: Characteristics of individuals according to differences in eGFR by cystatin C versus creatinine. Table S2: Associations of eGFR by cystatin C and creatinine with prevalent diabetes.
