# Supplementary material for: Association of Intraindividual Difference in Cystatin C and Creatinine Estimated Glomerular Filtration Rate With Diabetes
Source: J Diabetes Res. 2025 Nov 13;2025:9335243. doi: 10.1155/jdr/9335243 (PMC12634172; doi:10.1155/jdr/9335243)
Supplement: Supporting Information — Additional supporting information can be found online in the Supporting Information section. Table S1: Characteristics of individuals according to differences in eGFR by cystatin C versus creatinine. Table S2: Associations of eGFR by cystatin C and creatinine with prevalent diabetes. [file 9335243.f1.pdf]

Supplementary File

Association of intra-individual difference in cystatin c and creatinine estimated glomerular filtration rate with diabetes

Lingyu Zhang et. al

Table S1: Characteristics of individuals according to differences in eGFR by cystatin c versus creatinine

	Differences in eGFR by cystatin c versus creatinine			P value
	-15 ~ 15 (midrange)	< -15 (negative)	> 15 (positive)	
Participants, No.	8057 (67.9)	2699 (22.7)	1113 (9.4)	
Age, years				
Continuous	59.58(9.38)	63.61(9.73)	57.87(8.63)	<0.001
45-54	2841(35.3)	550(20.4)	462(41.5)	<0.001
55-64	2845(35.3)	944(35.0)	390(35.0)	
65-74	1802(22.4)	819(30.3)	224(20.1)	
75~	569(7.1)	386(14.3)	37(3.3)	
Female, n (%)	4213(52.3)	1892(70.1)	250(22.5)	<0.001
Married, n (%)	7126(88.4)	2240(83.0)	1016(91.3)	<0.001
Educational level, n (%)				<0.001
Primary or below	4443(55.1)	1820(67.4)	523(47.0)	
Middle or above	3614(44.9)	879(32.6)	590(53.0)	
BMI, kg/m2				
Continuous	23.90(3.67)	24.45(4.15)	23.54(3.18)	<0.001
<23.9	4259(52.9)	1251(46.4)	645(58.0)	<0.001
24-27.9	2647(32.9)	895(33.2)	358(32.2)	
≥28	1151(14.3)	553(20.5)	110(9.9)	
Smoking status, n (%)				<0.001
Never	4735(58.8)	1860(68.9)	482(43.3)	
Current	2264(28.1)	599(22.2)	414(37.2)	
Quit	1058(13.1)	240(8.9)	217(19.5)	
Current drinking, n (%)	2943(36.5)	618(22.9)	627(56.3)	<0.001

Data are presented as the mean (SD) or number (%), as appropriate.
Abbreviations: SD, standard deviation; BMI, body mass index; eGFR: estimated glomerular filtration rate
Unit of eGFR was ml/minute/1.73 m2 estimated by CKD-EPI equations

Table S2: Associations of eGFR by cystatin c and creatinine with prevalent diabetes

	Unadjusted		Adjusted	
	OR (95% CI)	P value	OR (95% CI)	P value
eGFRcr				
>=90	Ref			
60-89.9	0.822(0.723-0.933)	0.003	1.005(0.895-1.126)	0.931
<60	0.998(0.779-1.268)	0.984	1.381(1.093-1.731)	0.006
eGFRcys				
>=90	Ref			
60-89.9	1.226(1.111-1.352)	<0.001	0.927(0.83-1.036)	0.182
<60	2.105(1.755-2.516)	<0.001	1.345(1.093-1.651)	0.005

Abbreviation: OR, odds ratio; CI, confidence interval; eGFR: estimated glomerular filtration rate
Unit of eGFR was ml/minute/1.73 m2 estimated by CKD-EPI equations
The eGFRdiff was defined as the absolute difference between cystatin c and creatinine-based eGFR levels

Adjusted for age, sex, education degree, marriage status, smoking status, current drinking, BMI group and hypertension
